# Effects of Vitamin and Mineral Supplementation During Gestation in Beef Heifers on Immunoglobulin Concentrations in Colostrum and Immune Responses in Naturally and Artificially Reared Calves

**DOI:** 10.3390/vetsci11120635

**Published:** 2024-12-07

**Authors:** Jennifer L. Hurlbert, Friederike Baumgaertner, Kerri A. Bochantin-Winders, Isabella M. Jurgens, Kevin K. Sedivec, Carl R. Dahlen

**Affiliations:** 1 Department of Animal Sciences and Center for Nutrition and Pregnancy, North Dakota State University, Fargo, ND 58108, USA; 2 Central Grasslands Research Extension Center, North Dakota State University, Streeter, ND 58483, USA

**Keywords:** beef heifers, immune function, immunoglobulins, minerals, vaccination, vitamins

## Abstract

Two experiments assessed the effects of providing a vitamin and mineral supplement to gestating beef heifers on concentrations of immunoglobulins (Ig) in colostrum and calf serum 24 h after feeding maternal colostrum (Exp. 1) or a colostrum-replacement product (Exp. 2). Angus-based heifers (*n* = 31, Exp. 1; *n* = 14, Exp. 2) were fed a basal diet (CON) or were fed a basal diet plus a vitamin and mineral supplement (VTM) from breeding (Exp. 1) or 60 d pre-breeding (Exp. 2) through calving. Colostrum was collected at calving, and serum was collected from calves 24 h after colostrum consumption to evaluate passive transfer. Serum was collected from calves in Exp. 1 to determine serum titers in response to vaccination at birth, pasture turn out, weaning, and 14 d after vaccination. Concentrations of IgG, IgM, or IgA in colostrum or in calf serum at 24 h were not impacted by dam treatment (*p* ≥ 0.21); however, concentrations of Ig in calf serum at 24 h were greater (*p* ≤ 0.01) in calves receiving maternal colostrum than those receiving a colostrum replacer. Calves born to VTM heifers had greater antibody titers at birth, pasture turn out, and weaning for infectious bovine rhinotracheitis (IBR), bovine viral diarrhea virus type 2 (BVD-2), and bovine respiratory syncytial virus (BRSV), respectively. Our results suggest that the programming of immune function in calves via prenatal nutrition appears to extend postnatally in CON and VTM offspring.

## Simple Summary

The nutritional management of pregnant females can have lasting impacts on offspring growth, health, and well-being during postnatal life. Providing supplemental vitamins and minerals during pregnancy may influence offspring development and immune function. In two experiments, we investigated the impacts of supplementing vitamins and minerals to pregnant beef heifers on immunological components of maternal colostrum and resultant calf immunoglobulins in serum after consuming maternal colostrum or a colostrum replacement product. Antibody titers in blood from calves were also evaluated in response to receiving vaccinations through weaning. We observed no influence of maternal supplementation on colostral immunoglobulins or calf serum immunoglobulins 24 h after consuming colostrum, but calves receiving colostrum from their dams had greater immunoglobulin concentrations in their serum compared with calves receiving a colostrum replacer. Additionally, calves born to vitamin and mineral supplemented dams had increased titer responses to vaccination at weaning compared with calves born to non-supplemented dams. These data indicate that supplementary vitamins and minerals to gestating beef heifers may be a critical factor in programming offspring immune system development, ensuing animal health, and supporting profitability in beef operations.

## 1. Introduction

In beef cow diets, vitamins and trace minerals are required for numerous aspects of production, including reproduction, growth and performance, immune function, and overall health [1,2,3]. In pregnant cows and heifers, several minerals are transferred across the maternal–fetal interface to the developing fetus for normal growth demands and for the establishment of a postnatal mineral reserve [4,5]. Previous research models in our laboratory have explored the relationship between maternal vitamin and mineral supplementation and/or maternal rate of gain during the first trimester of pregnancy or throughout gestation on offspring mineral status, circulating metabolic and endocrine profiles, performance, transcriptomic profiles of key metabolic tissues, microbial colonization of the neonate, and puberty attainment in offspring raised as replacement heifers [5,6,7,8]. Briefly, these efforts have shown improved liver concentrations of Se, Cu, Co, and Zn in calves at birth, altered serum concentrations of proinflammatory cytokines, and enhanced postnatal performance (17.5 kg body weight (BW) advantage) through 15 months of age in offspring from dams supplemented with vitamins and minerals throughout gestation compared with a non-supplemented cohort [5,7,9].

Several minerals serve critical roles as structural components in several enzymes related to oxidative stress and in the scavenging and inactivation of reactive oxygen species, cytokine production for the establishment of innate and acquired immunity, mitochondrial energy production, acute-phase protein responses to stress, inflammatory responses, and antibody responses to vaccination, to name a few [10]. Nutrient-deficient diets provided to gestating cows and heifers have shown to increase corticosteroids in calves, such as cortisol, which can reduce metabolic rates, vigor, and postnatal calf survival [11]. Supplementing vitamins and minerals and meeting nutrient requirements in daily feed deliveries to the gestating dam may minimize the negative effects of stress on both maternal and postnatal calf immune systems, especially during critical management events throughout pre-weaning development (handling, weaning, vaccination, etc.) [10]. However, this largely unexplored concept of prenatal vitamin and mineral supplementation with regard to the pregnant dam and the subsequent impacts on postnatal health and immune function in their offspring warrants future investigation.

The health of beef calves, established early in life from the maternal transfer of antibodies and postnatal vaccination, is imperative for the well-being, productivity, and economics of cow–calf enterprises [12,13]. Calves depend on adequate maternal antibody transfer through immunoglobulins (Ig) in colostrum as the first line of defense for environmental stressors and disease exposure early in life, as the bovine placenta prevents efficient transfer of Ig to the fetus [10,12,14]. Successful passive transfer of immunity by the ingestion of maternal colostrum of adequate quality and quantity or a colostrum-replacement product to the neonate has been a topic of interest in dairy calves for decades [15,16,17], but fewer studies highlight the establishment of both the innate and adaptive immune systems in beef calves. Although several authors have described the effects of postnatal vitamin and mineral nutrition on immune responses to vaccination, environmental stressors, or other immune challenges in beef cattle during the growing and finishing periods [18,19], the mechanisms of programmed immune function in calves via maternal nutritional status during gestation is unclear [2]. Enhancing calf immunity during the postnatal period with prenatal vaccination of the dam and postnatal vaccination of the calf and potentially long-term effects of maternal programming during gestation may reduce the incidence of disease, which can minimize costs of medication and labor and increase average daily gains and potentially improve carcass quality. Certainly, reducing treatment costs, maintaining calf health, and reducing disease mortality through weaning by providing strategic management to the gestating dam could be critical for the cow–calf producer’s bottom line and warrants careful consideration.

Therefore, our objectives were to determine the effects of supplementing beef heifers with vitamins and minerals during gestation on concentrations of Ig in colostrum, the passive transfer of immunity in naturally and artificially reared calves, and postnatal antibody responses to vaccination. We hypothesized that supplementing beef heifers with vitamins and minerals during gestation will impact colostrum concentrations of Ig, the subsequent passive transfer of immunity, and later postnatal immune responses in calves born to vitamin- and mineral-supplemented dams compared with a non-supplemented cohort. Our results suggest that prenatal vitamin and mineral supplementation did not affect concentrations of Ig in colostrum of the dam at calving or in the serum of calves 24 h after colostrum consumption. However, calves suckling their dams had greater concentrations of Ig in serum compared with calves receiving a colostrum replacer. Finally, maternal vitamin and mineral supplementation appeared to influence calf postnatal antibody titer responses to vaccination through weaning. The results of the current study warrant further investigation into tissue-level fetal programming responses to gestational vitamin and mineral supplementation that may further implicate prenatal dam nutrition with postnatal calf health and well-being.

## 2. Materials and Methods

### 2.1. Animals, Housing, and Diet

Two experiments were conducted to evaluate the effects of providing a vitamin and mineral supplement to beef heifers throughout gestation on concentrations of immunoglobulin (Ig) in colostrum and in calf serum 24 h after the consumption of maternal colostrum (Exp. 1) or a colostrum replacer (Exp. 2). Complete descriptions of the animals, facilities, and feed ingredients used in Exp. 1 and 2 have been previously characterized [5,9]. Briefly, Angus-based heifers (*n* = 72, 14 to 15 months of age, initial BW = 380.4 ± 50.56 kg (SD throughout methods)) were managed in an individual feeding system (American Calan; Northwood, NH) at the NDSU Animal Nutrition and Physiology Center (ANPC; Fargo, ND). Heifers were randomly assigned to receive either a basal diet targeting a gain of 0.45 kg·heifer^−1^·day^−1^ (CON; *n* = 36) or a basal diet plus a loose product vitamin and mineral supplement (Table 1; Purina Wind and Rain Storm All Season 7.5 Complete, Land O’Lakes, Inc., Arden Hills, MN, USA) top-dressed on the total mixed ration (TMR) at a rate of 113 g·heifer^−1^·day^−1^ (VTM; *n* = 36), as per the company-directed feeding rate. All heifers were subjected to a 7-day Select Synch + CIDR estrus synchronization protocol and bred via artificial insemination (AI) to female-sexed semen from a single sire. Transrectal ultrasonography was conducted on d 35 and 65 to evaluate the presence of a fetus and to confirm the presence of a female calf, respectively.

### 2.2. Experiment 1

For Exp. 1, dietary treatments for F0 dams began at breeding. Heifers becoming pregnant with female fetuses after first-service AI (CON, *n* = 14; VTM, *n* = 17) were transported to the NDSU Beef Cattle Research Complex (BCRC; Fargo, ND, USA), adapted to the Insentec Roughage Intake Control Feeding System (Hokofarm B.V., Marknesse, The Netherlands), and dietary treatments (CON or VTM) were continued throughout gestation. The diet composition and delivery strategies for both Exp. 1 and Exp. 2 have been previously outlined [5,9]. Heifers pregnant with female fetuses remained on their respective dietary treatments through calving. All heifers were weighed every 2 weeks, and feed deliveries were adjusted on a biweekly basis to achieve target BW gains of 0.45 kg·heifer^−1^·day^−1^, with feed deliveries adapted to provide ad libitum feed intakes from d 214 of pregnancy through calving.

At calving, all female calves and their dams were separated immediately for a brief period to perform pre-suckling blood and colostrum collections. For both experiments, blood samples were collected from calves pre-suckling (within 2 h of birth) and 24 h after via jugular venipuncture into 10 mL serum vacutainer tubes (Becton Dickinson Co., Franklin Lakes, NJ, USA) for the evaluation of Ig concentration in serum prior to and 24 h after colostrum consumption. Blood samples were allowed to clot and were placed on ice until centrifugation. Samples were centrifuged at 1500× *g* at 4 °C for 20 min, aliquoted into 2 mL plastic microtubes, and stored at −20 °C until laboratorial analysis.

Samples of colostrum from the dam at calving were obtained by milking the rear-right quarter of the udder completely using a portable milk machine (InterPuls, Albinea, Italy) for both experiments. Similar milking methods were used across several experiments to provide consistency across studies in our laboratory. Dams were administered 1 mL of oxytocin (20 IU) immediately prior to collection to stimulate colostrum letdown. Then, the teat was cleaned with an iodine dip and stripped 3 times before attaching the milk machine to the teat to ensure flow, and the udder was massaged during collection to provide stimulation and complete emptying of the quarter. Hand milking was also performed after colostrum flow ceased with a milking machine. Colostrum mass and volume were recorded by emptying the collected colostrum from the portable milking machine, weighing the colostrum on a bench-top scale, and measuring the volume with a 1000 mL graduated cylinder [5]. Samples of colostrum were aliquoted in 15 mL plastic conical tubes and stored at −20 °C until analysis.

Calves and cows were then rejoined in maternity pens for 24 h and observed to ensure that the calf consumed the maternal colostrum within 2 h of birth. At 24 h after first colostrum consumption, blood was collected and vaccinations (Table 2) were administered in the neonatal calves for protection against respiratory viruses (infectious bovine rhinotracheitis (IBR), bovine parainfluenza-3 virus (PI-3), and bovine respiratory syncytial virus (BRSV)) and clostridial diseases. Blood was then collected 14 d after vaccination. This blood sampling scheme was relative to the day post-vaccination and took place over 6 key time points during pre-weaning development. Calf ages for blood collections to assess antibody titers were as follows: 24 h of age (day of vaccination) and 15 d of age; pasture turn out: 40.4 ± 3.73 d of age (day of vaccination) and 54.4 ± 3.73 d of age; and weaning: 184.4 ± 3.73 d of age (day of vaccination) and 198.4 ± 3.73 d of age. Vaccinations administered at pasture turn out (40.4 ± 3.73 d of age) included protection against respiratory viruses (IBR, PI-3, BRSV, bovine viral diarrhea virus type 1 (BVDV-1), bovine viral diarrhea virus type 2 (BCDV-2), and Mannheimia haemolytica), clostridial diseases, and pinkeye. At 7 d prior to weaning (184.4 ± 3.73 d of age), calves received vaccinations to protect against respiratory viruses (IBR, PI-3, BRSV, BVDV-1, BVDV-2, and Mannheimia haemolytica); clostridial diseases; and pinkeye. Blood samples were handled and stored according to the methods described above. All calves and postpartum cows were managed to receive a common diet ad libitum containing the vitamin and mineral supplement after calving through d 191 ± 3.7 post-calving (weaning). Additional postnatal evaluations of F1 calves born in Exp. 1 have been previously reported [5].

### 2.3. Experiment 2

For Exp. 2, heifers that did not become pregnant to first-service AI (CON, n = 19; VTM, *n* = 18) were re-synchronized for estrus and bred via AI 60 days after initial dietary treatments began. Diets were delivered, managed, and adjusted for dams confirmed pregnant with female fetuses using the same methods and timelines as described in Exp. 1 [5,9]. Heifer calves born (CON, *n* = 7; VTM, *n* = 7) were separated from their dams immediately after birth and relocated to individual pens, and blood collections were performed on the calves prior to colostrum consumption using the same methods as previously described. Colostrum was collected from the dam within 2 h of calving for the evaluation of concentrations of Ig, also using the methods described in Exp. 1, and then, dams were trained for a separate experiment involving the evaluation of daily milking in a parlor system at the NDSU Dairy Research and Teaching Unit [20]. All female calves in Exp. 2 were therefore not fed maternal colostrum but instead were provided 1.4 L of a plasma-derived colostrum replacer containing 150 g of a globulin protein (Lifeline Rescue High-Level Colostrum Replacer; APC, Inc.; Ankeny, IA, USA [9]) via an esophageal feeder within 2 h of birth followed by 2 L of a milk replacer (Duralife Optimal Non-Medicated Calf Milk Replacer; Duralife; Fort Worth, TX, USA) via an esophageal feeder 12 and 24 h after. At 24 h, blood was collected from calves to evaluate passive transfer of immunity. Calves were euthanized at 30 h for additional evaluations of organ morphometric characteristics and tissue micronutrient status [9].

### 2.4. Laboratorial Analysis: Concentrations of Ig

Concentrations of immunoglobulin G (IgG), M (IgM), and A (IgA) were quantified in colostrum and serum (pre-suckling and 24 h after) using bovine single radial immunodiffusion (RID) plate kits for assessing IgG (200 to 3000 mg/dL), IgM (50 to 400 mg/dL), and IgA (50 to 400 mg/dL) (Triple J Farms; Bellingham, WA, USA), according to the manufacturer’s instructions. Each RID kit consisted of one 24-well plate with an agarose gel containing an anti-bovine Ig antibody. The IgG, IgM, and IgA RID kits included three standard solutions of bovine reference sera: 280, 1400, and 2800 mg/dL; 62, 200, and 381 mg/dL; and 53, 194, and 386 mg/dL, respectively. Three additional standards were prepared for IgG, IgM, and IgA plates to establish a standard curve. Plate optimization was performed following previously used methods in our laboratory [21].

Briefly, plates were first optimized by plating kit standards, a pooled sample control (both colostrum and serum), and an unknown sample in duplicate to determine required sample dilutions. Samples were diluted as necessary in a 0.85% saline solution. Effective dilutions for determining colostrum IgG, IgM, and IgA were as follows: 1 to 8, 1 to 1, and 1 to 1, respectively. Effective dilutions for determining serum IgG, IgM, and IgA were 1 to 2, 1 to 2, and 1 to 1, respectively. After effective dilutions were determined, plates were prepared with the 6 standard reference sera and the pooled controls, in duplicate, at the beginning and end of each set of colostrum or serum samples for the respective run. Five µL of each standard, pooled control, and unknown sample was placed into the respective wells, and plates were covered and sealed in the original product bags to incubate in a Styrofoam container for 48 h at 23 °C. A digital caliper (62379-531, VWR, Radnor, PA, USA) was used to measure each precipitation ring diameter produced in each well three times at 48 h. Additional measurements were recorded if the CV was >5%. The average of the three measurements was used as the final reading for the respective well, and the squared average of the duplicate means was used to determine the mean concentration of IgG, IgM, or IgA in serum or colostrum. Colostrum and serum samples were plated in duplicate, and intra-assay CVs for IgG, IgM, and IgA plates for colostrum were 0.40%, 0.09%, and 4.1%, respectively. Intra-assay CVs for IgG, IgM, and IgA plates for serum were 1.72%, 3.35%, and 0.49%, respectively. The inter-assay CVs of the pooled controls for IgG, IgM, and IgA for colostrum were 2.18%, 2.13%, and 5.45%, respectively, and the inter-assay CVs of the pooled controls for serum IgG, IgM, and IgA were 11.57%, 9.66%, and 6.91%, respectively.

### 2.5. Laboratorial Analysis: Titer Response to Vaccination

Serum was analyzed at the Oklahoma State University Animal Disease Diagnostic Laboratory (OADDL; Stillwater, OK, USA) using serum neutralization for the detection of antibodies for BVDV type 1 and type 2, BRSV, IBR, and PI-3. Serum titers were reported as the log base 2 of the greatest dilution of serum that produced complete protection of the cells [21,22]. The lowest tested dilution for all antibodies (BVDV-1, BVDV-2, IBR, BRSV, and PI-3) was 1:4, and the greatest tested dilutions were 1:8192 for BVDV-1; 1:16,384 for BVDV-2; 1:1024 for IBR and BRSV; and 1:2048 for PI-3. According to the OADDL, samples with undetected antibodies at a 1:4 dilution were considered seronegative, and samples with detected antibodies at dilutions of >1:4 were considered seropositive.

### 2.6. Statistical Analysis

Data were analyzed for normality using the UNIVARIATE procedure of SAS 9.4 (SAS Inst. Inc., Cary, NC, USA). Log transformations were made for serum antibody titer data. Data were analyzed as a completely randomized design, with individual animal as the experimental unit. Colostrum immunoglobulin concentrations were analyzed using the MIXED procedure of SAS at a single point in time with main effects of treatment (CON vs. VTM) and group (Exp. 1 vs. Exp. 2). Concentrations of immunoglobulins in calf serum at 24 h were analyzed using the MIXED procedure, with repeated measures in time and main effects of maternal treatment (CON vs. VTM), calving group (Exp. 1 (suckling calves) vs. Exp 2 (tube-fed calves)), and the respective interaction. The CORR procedure was used to determine correlations between serum immunoglobulin concentrations in the calf at 24 h and concentrations of immunoglobulins in maternal colostrum (Exp. 1 only). Finally, data for serum neutralizing titers for calves (Exp. 1 only) were first transformed using a log2 transformation. Data were analyzed as repeated measures in time using the main effects of maternal treatment, day relative to vaccination, and the respective interaction. Covariance structures were tested where appropriate, and the structure with the lowest AIC fit statistic was selected for use in the final analysis. The PDIFF option of SAS was used with the Tukey–Kramer adjustment, and results were reported as least square means (LSMEANS) with their standard errors. Significance was determined at *p* ≤ 0.05, and tendencies were considered at 0.5 > *p* ≤ 0.10.

## 3. Results

### 3.1. Immunoglobulin Concentrations in Colostrum and Serum

Maternal vitamin and mineral supplementation throughout gestation did not impact (*p* ≥ 0.32) concentrations of IgG, IgM, IgA, or total Ig in colostrum in Exp. 1 or 2 (Table 3). All calves from both experiments had undetectable concentrations of IgG, IgM, and IgA at birth, which validated our pre-suckling collection method for other blood components described in the model experiments [5,9].

Calves born to CON and VTM dams in Exp. 1 had no differences (*p* ≥ 0.56) in serum concentrations of IgG, IgM, IgA, and total Ig between treatments at 24 h after colostrum consumption (Table 4). However, concentrations of IgG, IgM, and IgA in colostrum were positively correlated or tended to be correlated with concentrations of IgG (*p* = 0.10; r = 0.32); IgM (*p* = 0.01; r = 0.64); and IgA (*p* = 0.01; r = 0.49), in calf serum at 24 h of age in Exp. 1.

Calves born to CON and VTM dams in Exp. 2 also had no differences (*p* ≥ 0.21) in serum concentrations of IgG, IgM, or total Ig at 24 h of age (Table 4). Interestingly, calves from Exp. 2 had no detectable concentrations of IgA in serum 24 h after consuming a colostrum replacer (Table 4).

Although serum Ig concentrations in calves were not influenced by the interaction of treatment and calving group (*p* ≥ 0.43) or the main effect of treatment (*p* ≥ 0.78), we observed an effect of calving group (Exp. 1 vs. Exp. 2; *p* ≤ 0.01) on mean concentrations of IgG, IgM, IgA, and total serum Ig 24 h after colostrum consumption (Table 4). Calves from Exp. 1 receiving maternal colostrum had greater (*p* ≤ 0.01) 24 h serum concentrations of IgG, IgM, and IgA compared with calves from Exp. 2 receiving a colostrum replacer (Table 4).

#### Antibody Titer Response to Vaccination

No treatment by day interactions were detected (*p* ≥ 0.18) for antibody titer levels in response to vaccinations provided at birth, pasture turn out, or weaning in calves from Exp. 1 (Table 5). At birth, VTM calves had greater IBR titer levels (*p* = 0.03) compared with CON calves. Titers for BVD-1, BVD-2, IBR, and PI-3 decreased (*p* ≤ 0.03) and titers for BRSV tended to decrease (*p* = 0.07) from the day of vaccinations at birth to 15 d of age.

At pasture turn out, VTM calves had greater (*p* = 0.03) serum BVD-2 titers and tended (*p* ≤ 0.09) to have greater serum IBR and PI-3 titers compared with CON calves (Table 5). Serum PI-3 titers for calves decreased (*p* = 0.04) from d 40 to 54 in response to vaccination.

At weaning, VTM calves had greater (*p* = 0.01) serum BRSV titers compared with CON calves (Table 5), but no other differences (*p* ≥ 0.43) were detected between treatments for the other antibodies evaluated. A positive immune response to weaning vaccinations was evident, as titers for BVD-1, BVD-2, IBR, BRSV, and PI-3 increased (*p* ≤ 0.01) on d 184 compared with d 198.

## 4. Discussion

### 4.1. Immunoglobulins in Colostrum

Vitamins and minerals play critical roles in the antioxidant defense system and overall immune system function in livestock species [10,14,22,23,24]. During pregnancy in cattle, several minerals are transferred efficiently across the maternal–fetal interface to support fetal growth and development, energy and protein metabolism, hormone and DNA synthesis, and the establishment of a postnatal micronutrient reserve in the calf [5,25,26]. Providing supplemental vitamins and minerals to the dam during gestation may alter several components of colostrum, including immunoglobulins, interferons, cytokines, lymphocytes, and other nutritional components of colostrum that support the immune system and growth of the neonate [10,15,27]. Previous work from our laboratory suggests that providing supplemental vitamins and minerals to pregnant beef heifers can promote increased colostrum production and greater concentrations of trace minerals in colostrum [5]. An enhanced supply of micronutrients delivered through the colostrum and preset in liver and other tissue storage sites in the calf at birth may be impactful for the calf responding to extrauterine stressors and immune challenges during postnatal life [14]. Our group and others have also observed altered concentrations of several minerals in neonatal calves, including Co, Cu, and Zn, and an increased calf BW (≥17.5 kg advantage) at weaning and beyond in calves exposed to prenatal vitamin and mineral supplementation [5,28], which highlights the several production-relevant programming impacts of prenatal micronutrient nutrition on offspring productivity.

Studies of prenatal nutrition in beef cows and heifers have produced variable outcomes on colostrum immunoglobulin concentrations and passive transfer of immunity to the neonate. To our knowledge, the current study is one of the only evaluations of concentrations of Ig in colostrum in beef heifers provided with a vitamin and mineral supplement throughout gestation or no supplement. Although our previous results suggest that vitamin- and mineral-supplemented dams had greater trace mineral concentrations in colostrum at calving than non-supplemented controls [5], the results of the current report indicate that vitamin and mineral supplementation to beef heifers during gestation had little effect on the Ig concentrations in colostrum at parturition.

Several authors have reported that differing trace mineral sources (organic complex vs. inorganic) supplemented to beef cows during late gestation also had minimal effects on concentrations of IgG [29], IgA, and total concentrations of Ig in colostrum at calving [30]. Similar results have also been reported in dairy cattle, in which the trace mineral source had no effect on concentrations of IgG [31] or IgM [32] in colostrum. In addition, though late gestation protein/energy supplementation in beef cows increased the total colostrum production, no impacts on concentrations of colostrum IgG were observed [33].

Our lab previously detected no differences in colostrum IgG concentrations at parturition when managing pregnant beef heifers on a moderate or low rate of gain during the first 12 weeks of gestation [21], despite other reports of compromised colostrum IgG concentrations in beef cows as a result of early gestation nutrient restriction, which may have been driven by differences in cow BW and body condition [34]. Taken together, although some scenarios of prenatal nutritional insults appear to impact cow BW at calving and subsequent concentrations of immunoglobulins in colostrum [34], supplementing vitamins and minerals to beef heifers throughout gestation appeared to have little effect on the colostrum Ig evaluated herein. It is important to note that the nutritional management of the F0 dams in Exp. 1 and Exp. 2 in the current report initially described by our laboratory [5,9] was strategic, in that F0 dams were fed to achieve similar BW gains throughout pregnancy. Allowing differing rates of gain in VTM and CON dams from Exp. 1 and 2 may have affected the concentrations of immunoglobulins in colostrum [34]. Further investigating the immunological components of colostrum at calving and milk throughout lactation (e.g., lactoferrin, lymphocytes, and cytokines) from VTM and CON dams would allow for a more detailed evaluation of prenatal vitamin and mineral supplementation and contributions to calf growth and health in the postnatal period [10,12].

### 4.2. Passive Transfer of Immunity

Calves and other ruminants are born with a naïve, or agammaglobulinemic, immune system because of the inability of the syndesmochorial placenta of the cow to transport large protein molecules like immunoglobulins from the maternal to fetal circulation [12,17,35,36]. Although colostrum replacers can be effective when maternal colostrum is not available, there are several components of maternal colostrum, including proteins, lipids, carbohydrates, vitamins and minerals, immune factors, antimicrobial peptides, immune cells, endocrine factors, signaling molecules that modulate gene expression, and microorganisms, that all contribute to establishing the physiology of the immune system and the adaptations to extrauterine life in the calf [37]. Several of these components, however, are limited or omitted from colostrum replacers, which would otherwise promote nutrient absorption and Ig absorption in the first hours of life. Although some immunological responses can occur in the fetus to some antigens and pathogens [17], the newborn calf relies heavily on the passive transfer of Ig, immunity cells, and the nutritional components of colostrum soon after birth as the first line of defense to develop sufficient immunity before encountering postnatal stressors and synthesizing their own antibodies [12,15]. Successful passive transfer of immunoglobulins (IgG in serum < 10 g/L in dairy calves [12,16]) from colostrum helps to counteract the negative impacts of stress during extrauterine life of the calf, which can improve performance and vitality during postnatal development [13,38]. Several reports have shown that supplementing differing sources of trace minerals (inorganic minerals in sulfate forms vs. organic minerals complexed as proteinates) to pregnant dams [30] or providing trace-mineral-enriched feeds directly to calves [39,40] can be beneficial for the postnatal calf immune system.

However, in addition to observing no impacts of maternal vitamin and mineral supplementation on Ig concentrations in colostrum at calving, gestational supplementation of VTM had no observable effect on concentrations of IgG, IgM, IgA, or total Ig in naturally or artificially reared calves at 24 h. It is clear that the Ig in maternal colostrum was moderately correlated with the circulating Ig in the calf after colostrum consumption. It is evident, by an effect of the calving group (Exp. 1 vs. Exp. 2), that calves allowed to suckle their dams had a greater passive transfer of Ig compared with calves receiving a colostrum replacer. Calves receiving a maternal colostrum source in Exp. 1, on average, had serum concentrations of IgG, IgM, and total Ig at 24 h that were approximately 1.6-, 7.9-, and 1.6-fold greater than calves receiving a colostrum replacer in Exp. 2. Interestingly, unlike the calves in Exp. 1, none of the calves evaluated in Exp. 2 had detectable serum concentrations of IgA at 24 h of age, suggesting that the colostrum-replacement product used in Exp. 2 contained either no IgA or minimal concentrations of IgA, falling below the detection level of the assay.

There are several factors that influence passive transfer of immunity from the colostrum source to the neonate, including not only concentrations of Ig in the colostrum but also the feeding volume, time of consumption relative to birth, and microbial load in the colostrum [16]. In dairy calves, failure of passive transfer is considered when calves have serum IgG concentrations of <10 g/L between 24 and 48 h of life [12,16]. In general, acceptable passive transfer has been defined for calves with serum concentrations of IgG > 10 g/L between 24 and 48 h, but calves with serum IgG concentrations between 10 g/L and 24 g/L are considered to be at a greater risk for disease and morbidity after birth [12,16]. In the current study, only 7.14% of the calves (3.57% CON and 3.57% VTM) from Exp. 1 had serum concentrations of IgG less than 10 g/L at 24 h, indicating a failed passive transfer. Additionally, 92.9% of the neonates (42.9% CON and 50.0% VTM) in Exp. 1 had serum IgG concentrations of >10 g/L, suggesting an acceptable passive transfer of IgG. Of the 92.9% of calves with concentrations of >10 g/L, 39.3% of calves (21.4% CON and 17.9% VTM) had serum concentrations of IgG between 10 g/L and 24 g/L, and 53.5% of calves (21.4% CON and 32.1% VTM) had serum concentrations of IgG >24 g/L at 24 h.

Conversely, 7.1% of the calves (0% CON and 7.1% VTM) in Exp. 2 had serum concentrations of IgG of <10 g/L at 24 h, suggesting a failed passive transfer of immunity. However, 92.8% of calves (50.0% CON and 42.8% VTM) had serum concentrations of IgG between 10 g/L and 24 g/L at 24 h after suckling, but no calves had concentrations of serum IgG of >24 g/L at that time. Although serum concentrations of Ig in the neonate were not influenced by the maternal diet in the current report, it is clear that the colostrum source (maternal vs. replacement) was impactful on Ig absorption and circulating concentrations of Ig in the calf at 24 h of age. Certainly, several other factors besides gross Ig concentrations in colostrum can influence passive transfer of immunity in neonates.

Some reports have highlighted the role of the presence of the dam, cow–calf bonding, and natural behavioral elements of the suckling calf in relation to passive transfer in the immediate postnatal period, suggesting that calves allowed to nurse their dams have a greater rate of successful passive transfer compared with calves removed from their dams immediately [41,42]. Several others have compared tube feeding and teat-bottle feeding and the subsequent passive transfer of immunity, in which tube-fed calves had lower serum IgG concentrations after colostrum consumption compared with bottle-fed calves [11], which may be comparable to the tube-feeding and natural suckling methods of the current report. This theory is that there may be a greater efficiency of absorption of Ig for bottle-fed vs. tube-fed calves, as the esophageal groove is not activated with tube feeding as it is with calves expressing natural suckling behavior. Therefore, colostrum deposits directly into the reticulorumen with tube feeding as opposed to the omasum and abomasum with natural suckling [43,44]. It takes the rumen between 2 and 4 h to clear the colostrum to the small intestine, at which time the intestinal epithelium has started to mature, which reduces the number of intestinal cells actively absorbing Ig [11]. The absorptive capacity of Ig in intestinal cells starts to decrease from 6 to 12 h after birth through 48 h of age, reinforcing the importance of high-quality colostrum delivery to the calf immediately after birth [17]. The timeline from the colostrum feeding (~2 h after birth) to the first milk feeding (12 h later) in the hand-raised calves in Exp. 2 could certainly have impacted the absorptive capacity of Ig compared to the naturally reared calves in Exp. 1 that would have had constant access to colostrum/milk through 24 h of age. Calf stress can also play a role in absorptive capacities of Ig after birth, which can inhibit cortisol release by the neonate. Perhaps comparing circulating cortisol in the blood of Exp. 1 and Exp. 2 calves could indicate whether the maternal separation at birth contributed to neonatal corticosteroid levels and the subsequent absorptive capacity of Ig [17], which could also be critical for calf responses to environmental pathogen exposure in the pre-weaning period of development.

It is critical to note that calves in Exp. 2 were only fed one dose of the colostrum replacer at birth, followed by a milk replacer throughout the 24 h, while calves in Exp. 1 were allowed to suckle from their dams throughout the 24 h blood collection and beyond and were only limited by their dam’s level of production. Several authors have reported that when providing a plasma-derived colostrum replacer with ≥100 g/dose of IgG, feeding 2 or more doses of high-IgG colostrum replacers can help increase IgG intake and elevate the 24 h Ig concentrations in calf serum, therefore reducing the populations of calves with a failed passive transfer [45]. Therefore, while a known volume and concentration of colostrum was delivered to the calves in Exp. 2, serum Ig concentrations in the calves from Exp. 2 could have certainly been limited by the total volume of colostrum delivered and the method of colostrum delivery (tube vs. teat).

In a previously reported model experiment [5], we measured the total volume of colostrum produced from a single quarter at calving in dams from Exp. 1. By grossly estimating the total colostrum in the udder at calving, we can assume that VTM dams had approximately 1410 mL of colostrum on average at parturition, and CON dams had approximately 941 mL of colostrum. Although this does not account for colostrum production over the next 24 h in the dams from Exp. 1 and calf consumption of transition milk, it does provide a gross comparison with the total colostrum delivered to the calves in Exp. 2 (1400 mL). Although the total volume of colostrum delivery may have contributed some differences in concentrations of IgG, IgM, IgA, and total Ig in the calves in Exp. 1 and 2, we determined Ig concentrations in the colostrum replacer using the RID assay and found that the product contained 9494 ± 77.8 mg/dL of IgG, 609 ± 16.6 mg/dL of IgM, and undetectable concentrations of IgA. Meanwhile, the average maternal colostrum Ig concentrations were 12,886 ± 1150.8 mg/dL of IgG, 367 ± 25.2 mg/dL of IgM, and 288 ± 25.7 mg/dL of IgA. We suspect that it is more likely that the differences in average IgG, IgM, and IgA delivered through maternal colostrum, or the colostrum replacer explains the lesser serum Ig values reported for the calves in Exp. 2.

Taken together, these data suggest that the success of passive transfer in the calves from Exp. 1 benefited from the maternal colostrum having greater concentrations of Ig and being allowed to consume a greater volume of colostrum shortly after birth and, presumably, a greater total amount of Ig in colostrum within the first 24 h of birth. These results are representative of cow–calf production scenarios, where colostrum-replacement products are typically used as intervention strategies in scenarios of no/low dam colostrum production, poor dam temperament toward the calf, dystocia, or other scenarios that may limit the ability of the calf to successfully receive vital immunological components of colostrum. Unfortunately, little is actually known about the effectiveness of these strategies for beef calves [13,38], as compared to the well-established recommendations for dairy calves [16]. These data show that perhaps the colostrum-replacement product used in this experiment was a poor provider of Ig to the neonatal calf, and other products may be more or less successful in delivering adequate Ig to the calf. Nevertheless, evaluating the adaptive immune system, such as investigating titer responses to vaccination or immune challenges like transport and disease exposure, in beef calves receiving maternal or replacement colostrum sources would be informative for beef producers utilizing colostrum intervention strategies at birth.

### 4.3. Postnatal Antibody Response to Vaccination

Trace minerals are required during fetal growth for several developmental milestones related to the fetal nervous and immune systems that can ultimately impact postnatal offspring productivity [46,47]. Our results suggest that providing supplemental vitamins and minerals to beef heifers from conception to calving supported antibody titer responses to vaccination at critical time points during postnatal development. Specific antibodies affected at these time points included IBR, BVD-2, BRSV, and PI-3. Interestingly, each antibody titer evaluation at birth and at weaning was affected by day, indicating a lesser titer response 14 d after vaccination as opposed to the day of vaccination. An effect of day for PI-3 titer levels at pasture turn out and 14 d after vaccination showed a similar decreasing response; however, all titer levels measured at weaning indicated an enhanced titer response 14 d after vaccination as compared to the day of vaccination. These data suggest that while the timing of vaccination relative to a stressful event (e.g., transport, handling, and removal from the dam) may negatively impact an animal’s response to vaccination, it appears that the vaccinations provided 7 d prior to weaning allowed calves to have enhanced antibody titer levels 14 d later [48].

Although several studies investigate trace mineral supplementation in growing and finishing cattle and subsequent parameters of immune function [18,19], few studies evaluate vitamin and mineral supplementation during pregnancy in beef cattle and the programming of immune function in their offspring. Our lab recently reported that managing pregnant heifers on a moderate or low rate of gain during the first trimester of pregnancy did not affect antibody titers for BVD-1, BVD-2, BRSV, IBR, or PI-3 from birth through weaning in the offspring [21]. Additionally, a short-term energy restriction to 70% of daily NEm requirements in cows 40 days before calving has shown to reduce BVD-1 titers in offspring post-weaning, suggesting increased susceptibility to BVD-1 in offspring from energy-restricted dams compared with dams receiving 100% of energy requirements pre-calving [49]. Other authors have reported that supplementing vitamins and organic-complexed sources of minerals during late gestation in beef cows reduces the incidence of bovine respiratory disease (BRD) in their offspring during the growing period compared with offspring born to non-supplemented dams [28]. Taken together, several investigations of maternal nutrition during different stages of pregnancy—early [21], late [28,49,50], and the entire length of gestation [5,9]—indicate that nutritional perturbations to the dam during late gestation through calving may be more impactful on the fetal immune system and colostrogenesis compared with nutritional insults during early gestation.

Even so, the mechanisms by which maternal nutrition “programs” offspring immune status and postnatal susceptibility to disease is still largely unknown. Cows supplemented with vitamins and organic-complexed minerals during late gestation had calves that had a greater mRNA expression of superoxide dismutase (SOD), an enzyme comprising several minerals, which serves as a major antioxidant by inactivating reactive oxygen species (ROS) [10], at weaning compared with calves born to dams supplemented with inorganic mineral sources [50]. Interestingly, no treatment differences were detected in the mRNA expression of SOD in calves at birth, suggesting potential differences in cell oxidative damage, immune function, and susceptibility to disease later in postnatal life, dependent on postnatal environmental exposure [2,10,50]. Taken together, nutritional insults to the dam throughout pregnancy or during late gestation appear to program immune status and potential susceptibility to disease in the postnatal calf, although the mechanisms of immune system programming in the neonate are largely unknown. A warranted avenue of future investigation should focus on mineral-containing enzymes related to immune function and cell oxidative damage, including SOD and glutathione peroxidase (GPx). Because of the essentiality of minerals in the function of the aforementioned enzymes, perhaps the differing mineral status in dams and calves previously described in Exp. 1 and 2 [5,9] may be driving alterations observed postnatally in titer levels in response to vaccination. More specifically, dams from the CON treatment considered deficient or marginal for Cu and Se status throughout gestation described in Exp. 1 [5] may be responsible for the altered pre-weaning responses to vaccination and postnatal growth observed in VTM and CON calves.

Although calves in Exp. 1 and 2 were not considered deficient in any minerals at birth measured in the liver, muscle, or blood [5,9], a recent evaluation of neonatal jejunal tissue in calves from Exp. 2 revealed several differentially expressed genes specific to immune-related biological processes and pathways in VTM calves, including the cytokine–cytokine receptor interaction pathway [51]. Further evaluating the mRNA expression of genes related to immune function, SOD activity, GPx activity, and total antioxidant capacity in key tissues related to calf immune function and the humoral immune response in neonates evaluated in the current report may provide insights into the differential immune responses observed postnatally in the calves from Exp. 1. Specifically, investigating the mRNA expression of key contributors to immune system development and function (bone marrow, brown adipose, intestinal tissue and blood) collected from the harvested calves in Exp. 2 and measuring SOD and GPx activity in the blood from the calves in both experiments would help to further characterize calf immune status programmed by maternal vitamin and mineral supplementation to the dam during pregnancy [10,52,53].

## 5. Conclusions

Our results suggest that providing beef heifers with supplemental vitamins and minerals from pre-breeding through calving had minimal impacts on Ig concentrations in colostrum at parturition and in calf serum 24 h after birth within the parameters of the current experiment. However, calves that were naturally reared and received colostrum from their dams had a greater rate of successful passive transfer and greater concentrations of IgG, IgA, IgM, and total Ig in serum compared with calves removed from their dams and fed a colostrum replacer at birth. These results suggest that providing maternal colostrum is preferred over utilizing the colostrum-replacement product used in this experiment when colostrum intervention strategies are required at birth for beef calves. Certainly, the Ig composition and delivery recommendations of commercial colostrum-replacement products warrants further investigation.

Postnatal antibody titers in response to vaccination in calves at birth, pasture turn out, and at weaning were affected by prenatal vitamin and mineral supplementation in the dam, suggesting a programming effect of maternal diet to the neonatal immune system that may be supportive of postnatal performance, efficiency, and health. Improved performance and health are certainly major components of cow–calf operation productivity and efficiency, and reducing labor and treatment costs in the herd can be critical for cow and producer well-being. Future investigations should focus on the mRNA expression and antioxidant capacities of specific tissues related to immune function in calves, as insights gained from this future work would allow for further characterizations of immune function programming via maternal vitamin and mineral supplementation throughout gestation.

## Figures and Tables

**Table 1:** Company-guaranteed analysis of the vitamin and mineral supplement ^1^ provided to beef heifers during gestation in Exp. 1 and Exp. 2 ^2^.

Item	Assurance Levels	
Minerals	Min	Max
Ca, g/kg of DM	135.0	162.0
P, g/kg of DM	75.0	-
NaCl, g/kg of DM	180.0	216.0
Mg, g/kg of DM	10.0	-
K, g/kg of DM	10.0	-
Mn, mg/kg of DM	3600.0	-
Co, mg/kg of DM	12.0	-
Cu, mg/kg of DM	1200.0	-
I, mg/kg of DM	60.0	-
Se, mg/kg of DM	27.0	-
Zn, mg/kg of DM	3600.0	-
Vitamins, IU/kg of DM		
A	661,500.0	
D	66,150.0	
E	661.5	

^1^ Purina Wind and Rain Storm All Season 7.5 Complete Mineral (Land O’ Lakes, Inc., Arden Hills, MN, USA); ingredients: dicalcium phosphate, monocalcium phosphate, processed grain by-products, plant protein products, calcium carbonate, molasses products, salt, mineral oil, potassium chloride, magnesium oxide, ferric oxide, vitamin E supplement, vitamin A supplement, lignin sulfonate, cobalt carbonate, manganese sulfate, ethylenediamine dihydroiodide, zinc sulfate, copper chloride, vitamin D3 supplement, natural and artificial flavors, and sodium selenite. ^2^ Vitamin and mineral supplement (Table 1) fed at the company-directed rate of 113 g·heifer^−1^·day^−1^ from breeding to calving (Exp. 1) or from 60 d prior to breeding through calving (Exp. 2).

**Table 2:** Vaccinations administered to suckling calves in Exp. 1 from birth to weaning.

Timepoint ^1^	Vaccine Name	Antigens Included in Vaccine ^2^
**Birth**	Inforce 3 (Zoetis)	IBR, PI-3, and BRSV
	Ultrabac CD (Zoetis)	Clostridium perfringens types C and D
**Pasture Turn Out**	Inforce 3 (Zoetis)	IBR, PI-3, and BRSV
	Ultrabac 7/Somubac (Zoetis)	Clostridium chauvoei, Cl. septicum, Cl. novyi, Cl. sordellii, Cl. perfringens types C and D, and Haemophilus somnus
	One Shot BVD (Zoetis)	BVDV-1, BVDV-2, and Mannheimia haemolytica type A1
	Pinkeye (Addison Bio Labs)	Moraxella Bovoculi
**Weaning**	BoviShield Gold One Shot (Zoetis)	IBR, BVDV-1, BVDV-2, BRSV, PI-3, and Mannheimia haemolytica
	Ultrabac 7/Somubac (Zoetis)	Clostridium chauvoei, Cl. septicum, Cl. novyi, Cl. sordellii, Cl. perfringens types C and D, and Haemophilus somnus
	Pinkeye (Addison Bio Labs)	Moraxella Bovoculi

^1^ Calf ages at each key vaccination time point were as follows: birth (24 h of age), pasture turn out (40.4 ± 3.73 d of age), and weaning (184.4 ± 3.73 d of age). ^2^ Antigens evaluated in antibody titers were as follows: infectious bovine rhinotracheitis (IBR), bovine parainfluenza-3 virus (PI-3) bovine respiratory syncytial virus (BRSV), bovine viral diarrhea virus type 1 (BVDV-1), and bovine viral diarrhea virus type 2 (BCDV-2).

**Table 3:** Concentrations of immunoglobulin (Ig) G, M, and A in colostrum and total colostrum Ig concentrations in beef heifers assigned to receive a basal diet (CON) or a basal diet with the addition of a vitamin and mineral supplement ^1^ (VTM) during gestation.

	Treatment			
Concentration, mg/dL	CON	VTM	SE	*p*-Value
**Experiment 1 ^2^**				
IgG	12,769	13,003	1150.8	0.88
IgM	376.0	359.7	25.20	0.64
IgA	293.8	282.6	25.70	0.75
Total Ig	13,439	13,664	1173.6	0.89
**Experiment 2 ^3^**				
IgG	10,109	7482	1704.3	0.30
IgM	295.6	301.8	48.93	0.93
IgA	198.0	227.1	35.71	0.58
Total Ig	10,603	8011	1764.5	0.32

^1^ Vitamin and mineral supplement (Table 1) provided at the company-directed rate of 113 g·heifer^−1^·day^−1^ to VTM dams. ^2^ Heifers in Exp. 1 were assigned to dietary treatments (CON or VTM) at breeding and remained on respective treatments throughout pregnancy. Calves born to dams in Exp. 1 were allowed to suckle from their dam. ^3^ Heifers in Exp. 2 were assigned to dietary treatments (CON or VTM) 60 days prior to AI breeding and remained on respective treatments throughout pregnancy. Calves born to dams in Exp. 2 were separated from their dams at birth and fed a colostrum replacer within 2 h of birth and a milk replacer at 12 h and 24 h of age.

**Table 4:** Concentrations of immunoglobulin (Ig) G, M, and A in serum and total serum Ig concentrations in neonatal calves born to dams assigned to receive a basal diet (CON) or a basal diet with the addition of a vitamin and mineral supplement (VTM ^1^) during gestation.

	Experiment 1 ^2^		Experiment 2 ^3^					
	Treatment		Treatment			*p*-Values		
Concentration, mg/dL ^4^	CON	VTM	CON	VTM	SE	TRT	Calving Group ^5^	TRT × Calving Group
**IgG**								
24 h	2556.2	2630.5	1638.6	1582.7	417.44	0.98	0.01	0.86
**IgM**								
24 h	177.9	160.3	13.2	29.6	24.66	0.98	<0.001	0.43
**IgA**								
24 h	93.7	85.9	0.0	0.0	15.48	0.78	<0.001	0.78
**Total Ig**	2827.8	2907.3	1757.4	1722.3	496.45	0.96	0.01	0.89

^1^ Vitamin and mineral supplement (Table 1) provided at the company-directed rate of 113 g·heifer^−1^·day^−1^ to VTM dams. ^2^ Heifers in Exp. 1 were assigned to dietary treatments (CON or VTM) at breeding and remained on respective treatments throughout pregnancy. Calves born to dams in Exp. 1 were allowed to suckle from their dam. ^3^ Heifers in Exp. 2 were assigned to dietary treatments (CON or VTM) 60 days prior to AI breeding and remained on respective treatments throughout pregnancy. Calves born to dams in Exp. 2 were separated from their dams at birth and fed a colostrum replacer within 2 h of birth and a milk replacer at 12 h and 24 h of age. ^4^ Concentrations of Ig in the calf serum at 0 h (pre-suckling) were undetectable for all calves in both experiments, as expected, and only 24 h Ig concentrations in serum are shown above. ^5^ Calving group is the comparison between calves born in Exp. 1 (maternal colostrum ingested) vs. calves born in Exp. 2 (colostrum replacer ingested).

**Table 5:** Serum titers (log2 transformed) in postnatal beef calves with evaluation at 24 h of age, pasture turn out, and weaning.

Time Point	d 0 ^2^		d 14 ^1^			*p*-Values		
	CON	VTM	CON	VTM	SE	TRT	Day	TRT × Day
**Birth**								
Calf Age	24 h		15 d		-	-	-	-
BVD-12	8.42	8.63	6.67	7.47	0.471	0.25	0.01	0.50
BVD-2	9.50	9.59	8.75	8.41	0.467	0.77	0.03	0.62
IBR	3.92	4.59	3.33	3.59	0.231	0.03	0.01	0.33
BRSV	6.17	6.06	5.50	5.42	0.389	0.79	0.07	0.68
PI-3	7.17	7.24	5.67	6.18	0.417	0.45	0.01	0.57
**Pasture Turn out**								
Calf Age	40.4 ± 3.73		54.4 ± 3.73		-	-	-	-
BVD-1	5.58	5.24	5.33	5.47	0.442	0.34	0.22	0.53
BVD-2	6.00	7.00	5.67	6.53	0.451	0.03	0.34	0.87
IBR	2.25	2.41	2.17	2.41	0.123	0.07	0.71	0.71
BRSV	4.08	4.12	4.42	4.53	0.332	0.81	0.23	0.90
PI-3	4.67	5.06	3.83	4.53	0.349	0.09	0.04	0.64
**Weaning**								
Calf Age	184.4 ± 3.73		198.4 ± 3.73		-	-	-	-
BVD-1	2.25	2.59	3.55	3.41	0.281	0.68	<0.01	0.35
BVD-2	3.08	3.65	3.55	6.42	0.603	0.69	<0.01	0.52
IBR	2.00	2.00	6.27	5.88	0.276	0.43	<0.01	0.43
BRSV	2.00	2.47	2.36	6.65	0.33	0.01	0.01	0.18
PI-3	2.25	2.00	5.27	5.94	0.492	0.63	<0.01	0.30

^1^ Treatments of the F0 dams were as follows: VTM (*n* = 17): heifers received the basal diet plus the addition of a vitamin and mineral supplement from breeding through parturition; or CON (*n* = 14): heifers received a basal diet from breeding through parturition. ^2^ Serum neutralization analyses were conducted to determine antibody titer responses to vaccination for the detection of antibodies for bovine viral diarrhea virus (BVDV) type 1 and type 2, bovine respiratory syncytial virus (BRSV), infectious bovine rhinotracheitis (IBR), and parainfluenza 3 (PI-3) on the day of vaccination (d 0) and 14 d after vaccination. Means are reported as log-transformed values.

## Data Availability

Data are contained within this manuscript.

## References

[1] McDowell L.R. (1992). Minerals in Animal and Human Nutrition.

[2] Harvey K.M., Cooke R.F., Marques R.D.S. (2021). Supplementing Trace Minerals to Beef Cows during Gestation to Enhance Productive and Health Responses of the Offspring. Animals.

[3] Underwood E.J., Suttle N.F. (1999). The Mineral Nutrition of Livestock.

[4] Hidiroglou M., Knipfel J.E. (1981). Maternal-Fetal Relationships of Copper, Manganese, and Sulfur in Ruminants. A Review. J. Dairy Sci..

[5] Hurlbert J.L., Baumgaertner F., Menezes A.C.B., Bochantin K.A., Diniz W.J.S., Underdahl S.R., Dorsam S.T., Kirsch J.D., Sedivec K.K., Dahlen C.R. (2024). Supplementing vitamins and minerals to beef heifers during gestation: Impacts on mineral status in the dam and offspring, and growth and physiological responses of female offspring from birth to puberty. J. Anim. Sci..

[6] Diniz W.J.S., Reynolds L.P., Borowicz P.P., Ward A.K., Sedivec K.K., McCarthy K.L., Kassetas C.J., Baumgaertner F., Kirsch J.D., Dorsam S.T. (2021). Maternal Vitamin and Mineral Supplementation and Rate of Maternal Weight Gain Affects Placental Expression of Energy Metabolism and Transport-Related Genes. Genes.

[7] Luecke S.M., Holman D.B., Schmidt K.N., Gzyl K.E., Hurlbert J.L., Menezes A.C.B., Bochantin K.A., Kirsch J.D., Baumgaertner F., Sedivec K.K. (2023). Whole-body microbiota of newborn calves and their response to prenatal vitamin and mineral supplementation. Front. Microbiol..

[8] Menezes A.C.B., McCarthy K.L., Kassetas C.J., Baumgaertner F., Kirsch J.D., Dorsam S.T., Neville T.L., Ward A.K., Borowicz P.P., Reynolds L.P. (2022). Vitamin and Mineral Supplementation and Rate of Gain in Beef Heifers I: Effects on Dam Hormonal and Metabolic Status, Fetal Tissue and Organ Mass, and Concentration of Glucose and Fructose in Fetal Fluids at d 83 of Gestation. Animals.

[9] Hurlbert J.L., Menezes A.C.B., Baumgaertner F., Bochantin-Winders K.A., Jurgens I.M., Kirsch J.D., Amat S., Sedivec K.K., Swanson K.C., Dahlen C.R. (2024). Vitamin and mineral supplementation to beef heifers during gestation: Impacts on morphometric measurements of the neonatal calf, vitamin and trace mineral status, blood metabolite and endocrine profiles, and calf organ characteristics at 30 h after birth. J. Anim. Sci..

[10] Palomares R.A. (2022). Trace Minerals Supplementation with Great Impact on Beef Cattle Immunity and Health. Animals.

[11] Quigley J., Drewry J. (1998). Nutrient and Immunity Transfer from Cow to Calf Pre- and Postcalving. J. Dairy Sci..

[12] Weaver D.M., Tyler J.W., VanMetre D.C., Hostetler D.E., Barrington G.M. (2000). Passive transfer of colostral immunoglobulins in calves. J. Vet. Intern. Med..

[13] Waldner C.L., Rosengren L.B. (2009). Factors associated with serum immunoglobulin levels in beef calves from Alberta and Saskatchewan and association between passive transfer and health outcomes. Can. Vet. J..

[14] Kegley E.B., Ball J.J., Beck P.A., Bill E. (2016). Kunkle Interdisciplinary Beef Symposium: Impact of mineral and vitamin status on beef cattle immune function and health. J. Anim. Sci..

[15] Kertz A.F., Hill T.M., Quigley J.D., Heinrichs A.J., Linn J.G., Drackley J.K. (2017). A 100-Year Review: Calf nutrition and management. J. Dairy Sci..

[16] Godden S.M., Lombard J.E., Woolums A.R. (2019). Colostrum Management for Dairy Calves. Vet. Clin. N. Am.-Food Anim. Prac..

[17] Chase C.C.L., Hurley D.J., Reber A.J. (2008). Neonatal Immune Development in the Calf and Its Impact on Vaccine Response. Vet. Clin. N. Am.-Food Anim. Prac..

[18] Richeson J.T., Kegley E.B. (2011). Effect of supplemental trace minerals from injection on health and performance of highly stressed, newly received beef heifers. Prof. Anim. Sci..

[19] Galyean M.L., Malcolm-Callis K.J., Gunter S.A., Berrie R.A. (1995). Effects of Zinc Source and Level and Added Copper Lysine in the Receiving Diet on Performance by Growing and Finishing Steers. Prof. Anim. Sci..

[20] Baumgaertner F., Menezes A.C.B., Diniz W.J.D.S., Molden T., Hurlbert J.L., Bochantin-Winders K.A., Sedivec K.K., Wanchuk M., Kirsch J.D., Underdahl S.R. Effects of two different rates of body weight gain during the first trimester of pregnancy or supplementing vitamins and minerals throughout pregnancy on primiparous beef cow milk production and composition. Transl. Anim. Sci..

[21] Baumgaertner F., Menezes A.C.B., Diniz W.J.S., Hurlbert J.L., Bochantin K.A., Underdahl S.R., Kirsch J.D., Dorsam S.T., McCarthy K.L., Ramirez-Zamudio G.D. (2024). Effects of rate of body weight gain during the first trimester of gestation on beef heifer and offspring performance, concentrations of hormones and metabolites, and response to vaccination. J. Anim. Sci..

[22] Miqueo E., Mattioli G.A., Moore D.P., Bilbao M.G., Moran K.D., Relling A.E. (2024). Impact of Parenteral Maternal Supplementation with Trace Minerals and Vitamins on Neonatal Calf Antioxidant System and Growth in a Dairy Herd. Animals.

[23] Enjalbert F., Lebreton P., Salat O. (2006). Effects of copper, zinc and selenium status on performance and health in commercial dairy and beef herds: Retrospective study. J. Anim. Physiol. Anim. Nutr..

[24] Boland T.M., Brophy P.O., Callan J.J., Quinn P.J., Nowakowski P., Crosby T.F. (2005). The effects of mineral supplementation to ewes in late pregnancy on colostrum yield and immunoglobulin G absorption in their lambs. Livest. Prod. Sci..

[25] Abdelrahman M.M., Kincaid R.L. (1993). Deposition of Copper, Manganese, Zinc, and Selenium in Bovine Fetal Tissue at Different Stages of Gestation. J. Dairy Sci..

[26] Abdelrahman M.M., Kincaid R.L. (1995). Effect of Selenium Supplementation of Cows on Maternal Transfer of Selenium to Fetal and Newborn Calves. J. Dairy Sci..

[27] Ogilvie L., Van Winters B., Mion B., King K., Spricigo J.F.W., Karrow N.A., Steele M.A., Ribeiro E.S. (2023). Effects of replacing inorganic salts of trace minerals with organic trace minerals in the diet of prepartum cows on quality of colostrum and immunity of newborn calves. J. Dairy Sci..

[28] Marques R.S., Cooke R.F., Rodrigues M.C., Cappellozza B.I., Mills R.R., Larson C.K., Moriel P., Bohnert D.W. (2016). Effects of organic or inorganic cobalt, copper, manganese, and zinc supplementation to late-gestating beef cows on productive and physiological responses of the offspring. J. Anim. Sci..

[29] Muehlenbein E.L., Brink D.R., Deutscher G.H., Carlson M.P., Johnson A.B. (2001). Effects of inorganic and organic copper supplemented to first-calf cows on cow reproduction and calf health and performance. J. Anim. Sci..

[30] Price D.M., Arellano K.K., Irsik M., Rae D.O., Yelich J.V., Mjoun K., Hersom M.J. (2017). Effects of trace mineral supplement source during gestation and lactation in Angus and Brangus cows and subsequent calf immunoglobulin concentrations, growth, and development. Prof. Anim. Sci..

[31] Jacometo C.B., Osorio J.S., Socha M., Corrêa M.N., Piccioli-Cappelli F., Trevisi E., Loor J.J. (2015). Maternal consumption of organic trace minerals alters calf systemic and neutrophil mRNA and microRNA indicators of inflammation and oxidative stress. J. Dairy Sci..

[32] Kincaid R.L., Socha M.T. (2004). Inorganic versus complexed trace mineral supplements on performance of dairy cows. Prof. Anim. Sci..

[33] Kennedy V.C., Gaspers J.J., Mordhorst B.R., Stokka G.L., Swanson K.C., Bauer M.L., Vonnahme K.A. (2019). Late gestation supplementation of corn dried distiller’s grains plus solubles to beef cows fed a low-quality forage: III. effects on mammary gland blood flow, colostrum and milk production, and calf body weights. J. Anim. Sci..

[34] Noya A., Casasús I., Ferrer J., Sanz A. (2019). Long-term effects of maternal subnutrition in early pregnancy on cow-calf performance, immunological and physiological profiles during the next lactation. Animals.

[35] Menge C., Neufeld B., Hirt W., Schmeer N., Bauerfeind R., Baljer G., Wieler L.H. (1998). Compensation of preliminary blood phagocyte immaturity in the newborn calf. Vet. Immunol. Immunopathol..

[36] Hopkins B.A., Quigley J.D. (1997). Effects of Method of Colostrum Feeding and Colostrum Supplementation on Concentrations of Immunoglobulin G in the Serum of Neonatal Calves. J. Dairy Sci..

[37] Silva F.G., Silva S.R., Pereira A.M.F., Cerqueira J.L., Conceição C. (2024). A Comprehensive Review of Bovine Colostrum Components and Selected Aspects Regarding Their Impact on Neonatal Calf Physiology. Animals.

[38] Gamsjäger L., Haines D.M., Pajor E.A., Lévy M., Windeyer M.C. (2021). Impact of volume, immunoglobulin G concentration, and feeding method of colostrum product on neonatal nursing behavior and transfer of passive immunity in beef calves. Animal.

[39] Hall J.A., Bobe G., Vorachek W.R., Gorman M.E., Mosher W.D., Pirelli G.J. (2013). Effects of Feeding Selenium-Enriched Alfalfa Hay on Immunity and Health of Weaned Beef Calves. Biol. Trace Elem. Res..

[40] Hall J.A., Bobe G., Vorachek W.R., Estill C.T., Mosher W.D., Pirelli G.J., Gamroth M. (2014). Effect of supranutritional maternal and colostral selenium supplementation on passive absorption of immunoglobulin G in selenium-replete dairy calves. J. Dairy Sci..

[41] Selman I.E., McEwan A.D., Fisher E.W. (1971). Studies on dairy calves allowed to suckle their dams at fixed times post partum. Res. Vet. Sci..

[42] Haines D.M., Godden S.M. (2011). Short communication: Improving passive transfer of immunoglobulins in calves. III. Effect of artificial mothering. J. Dairy Sci..

[43] McGee M., Earley B. (2019). Review: Passive immunity in beef-suckler calves. Animal.

[44] Godden S.M., Haines D.M., Konkol K., Peterson J. (2009). Improving passive transfer of immunoglobulins in calves. II: Interaction between feeding method and volume of colostrum fed. J. Dairy Sci..

[45] Quigley J.D., Strohbehn R.E., Kost C.J., O’Brien M.M. (2001). Formulation of colostrum supplements, colostrum replacers and acquisition of passive immunity in neonatal calves. J. Dairy Sci..

[46] Hostetler C.E., Kincaid R.L., Mirando M.A. (2003). The role of essential trace elements in embryonic and fetal development in livestock. Vet. J..

[47] Van Emon M., Sanford C., Mccoski S. (2020). Impacts of Bovine Trace Mineral Supplementation on Maternal and Offspring Production and Health. Animals.

[48] Cortese V.S., Woolums A.R., Karisch B.B., Short T.H., Thoresen M., Badial P. (2020). Systemic and local immune responses of weaned beef calves vaccinated post-transportation and at the time of a mild respiratory tract infection. Bov. Pract..

[49] Moriel P., Piccolo M.B., Artioli L.F.A., Marques R.S., Poore M.H., Cooke R.F. (2016). Short-term energy restriction during late gestation of beef cows decreases postweaning calf humoral immune response to vaccination. J. Anim. Sci..

[50] Harvey K.M., Cooke R.F., Colombo E.A., Rett B., De Sousa O.A., Harvey L.M., Russell J.R., Pohler K.G., Brandão A.P. (2021). Supplementing organic-complexed or inorganic Co, Cu, Mn, and Zn to beef cows during gestation: Physiological and productive response of cows and their offspring until weaning. J. Anim. Sci..

[51] Craner A.J., Dahlen C.R., Hurlbert J.L., Menezes A.C.B., Banerjee P., Baumgaertner F., Bochantin-Winders K.A., Amat S., Sedivec K.K., Swanson K.C. (2024). Early life programming of the neonatal bovine jejunum in response in response to maternal vitamin and mineral supplementation. J. Dev. Orig. Health Dis..

[52] Maddox J.F., Aherne K.M., Reddy C.C., Sordillo L.M. (1999). Increased neutrophil adherence and adhesion molecule mRNA expression in endothelial cells during selenium deficiency. J. Leukoc. Biol..

[53] Keogh K., Kelly A.K., Kenny D.A. (2021). Effect of plane of nutrition in early life on the transcriptome of visceral adipose tissue in Angus heifer calves. Sci. Rep..

